# Internalized Evolutionary Inertia on Body Plan Evolution

**DOI:** 10.1111/ede.70041

**Published:** 2026-05-02

**Authors:** Wenxin Zeng, Naoki Irie

**Affiliations:** ^1^ Department of Biological Sciences, Graduate School of Science The University of Tokyo Tokyo Japan; ^2^ Research Center for Integrative Evolutionary Science, SOKENDAI Hayama Kanagawa Japan

**Keywords:** developmental bias, evolution, hourglass model, pleiotropic constraints

## Abstract

Despite the phenotypic diversity of animal species, the basic anatomical features, or body plan, of each animal phylum have been strictly conserved since their initial establishment in the early Cambrian. While this remarkable conservation could be explained by the conservation of the mid‐embryonic phase (the developmental hourglass model) when the body plan is established, the underlying evolutionary mechanisms remained largely unclear. In this respect, recent studies have highlighted intrinsic properties in development, such as robustness, stability, and pleiotropic constraints, as potential contributors to its limitation of phenotypic diversifications. These findings suggest a potential mechanism of how phenotypic evolution is intrinsically limited or biased. In this review, potential developmental factors that contributed to the intrinsic limiting effects of animal embryogenesis against phenotypic diversification will be overviewed, with a particular focus on the general relationship between evolution and developmental processes.

## Summary

This review examines how the developmental hourglass‐like conservation can potentially be explained by intrinsic developmental biases such as developmental robustness and stability.

## Graphical abstract

Animal development appears to intrinsically bias phenotypic evolution, though the exact mechanism is unclear. This review discusses potential mechanisms by which animal embryogenesis limits phenotypic diversification and considers whether inherent evolutionary inertia could allow us to predict future evolutionary paths.

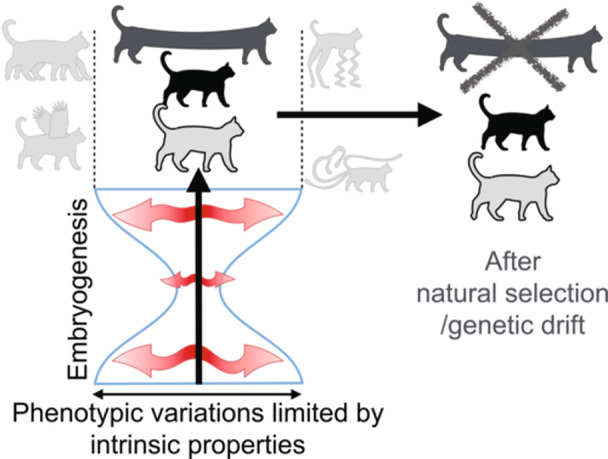

## 1 Introduction

The evolutionary process of organisms is basically explained by the emergence of phenotypic variations, primarily arising from genetic mutations and phenotypic plasticity (Price et al. 2003), followed by natural selections and other dynamic effects as studied in population genetics, such as genetic drift (Ohta 2011). In theory, this implies that evolution could generate an almost limitless range of phenotypes, provided that sufficient mutations occur and appropriate selection pressures act upon them. However, phenotypes that are deleterious or lethal must be excluded from the theoretical variation pool of phenotypes into which organisms can evolve. This includes phenotypes that prevent successful embryogenesis, as they would preclude reproductive success and thus be evolutionarily inviable. This can mean that phenotypic evolution is not completely free to proceed in all hypothetical directions (Raup 1966), and embryogenesis itself acts as one of the factors that bias the outcome of phenotypic evolution. This phenomenon is often referred to as developmental bias (Arthur 2004), though its distinction from related concepts, such as developmental constraints (Smith et al. 1985), developmental burden (McAllister 1981; Fujimoto et al. 2022), external (environment‐dependent) selection and internal (environment‐independent) selection (Fusco 2001), as well as a quantification of its significance compared with other factors in shaping evolution, still remains under debate (Hallgrímsson et al. 2002; Furusawa and Irie 2020). Despite these confusions, the consensus here is that embryogenesis is not only a source of diverse phenotypes but is also biasing or limiting phenotypic diversification (Arthur 2001).

**Figure 1:**
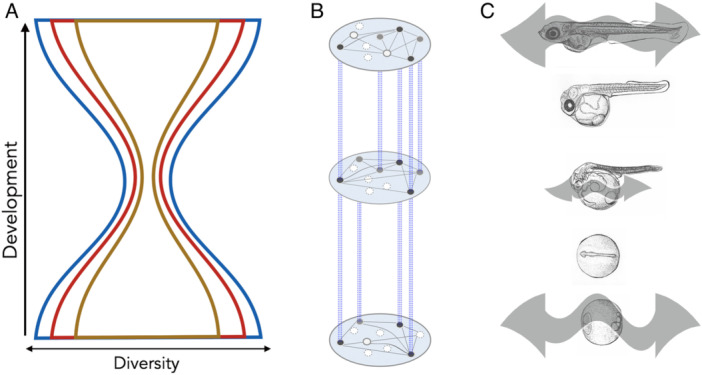
Features and tendencies during embryogenesis to explain body plan conservation. (A) The developmental hourglass model (Duboule 1994) explains the evolutionary diversity observed during embryogenesis. The bottleneck in the nested hourglasses represents that the mid‐embryonic period has always been the target of conservation ranging from intra‐species (Zalts and Yanai 2017) to larger evolutionary scales, such as phylum (Raff 1996; Hu et al. 2017). Additionally, this conserved period is predicted to define the body plan for each phylum (Rudolf 1996; Hu et al. 2017). Different colored hourglasses indicate different evolutionary scales. (B) Genes involved in various biological processes (pleiotropically expressed genes, represented by dark circles) are enriched during the conserved mid‐embryonic period (Hu et al. 2017). Dashed blue lines indicate that the genes are commonly expressed across different developmental stages. Of note, highly pleiotropically expressed genes tended to exhibit lethal phenotypes and were predicted to have more protein–protein interactions (Hu et al. 2017). (C) The conserved mid‐embryonic phase is not only robust against genetic mutations and environmental perturbations (Uchida et al. 2018) but also shows greater developmental stability (Uchida et al. 2022) against stochastic noise than earlier and later stages. This suggests that the conserved mid‐embryonic phase has a lower potential to generate phenotypic variation, which may contribute to reduced evolutionary diversity. The size of the double‐headed arrows indicates the extent of phenotypic variation, with larger arrows representing greater variability. [Color figure can be viewed at wileyonlinelibrary.com]

Classically, arguments by pioneering scientists such as Karl Ernst von Baer and Ernst Haeckel led to the idea that the earlier embryonic stages tend to be more evolutionarily conserved (von Baer 1828; Haeckel 1866). Notably, von Baer himself did not accept the concept of evolution, and both researchers lacked the technology to observe early embryos as precisely as is possible today, yet the idea made a substantial impact on biology. The essence of this idea was further expanded by subsequent researchers, who argued that the earliest embryonic phase is the biggest biasing step toward evolutionary diversification (Wimsatt 1986; Rasmussen 1987). In contrast, Duboule (1994) claimed that it is the mid‐embryonic, or body plan‐establishing phase (the phylotypic period (Sander 1983; Richardson et al. 1998)), where the most conserved, and presumably highly biasing stage appears, proposing the developmental hourglass model (Figure 1A). However, validating these hypotheses has remained a long‐standing challenge. This is because identifying conserved developmental stages is problematic, and elucidating the evolutionary mechanisms that limit diversification is even more difficult. For example, comparative morphological analyses have faced difficulties in testing these hypotheses as the quantification of evolutionary conservation by comparing qualitatively different features (e.g., diversities in egg sizes vs. those in somite numbers) is problematic (Richardson et al. 1997; Bininda‐Emonds et al. 2003), which led to the exclusion of early and late developmental stages in comparison. Some studies have circumvented this issue by analyzing properties of genes (e.g., evolutionary origin, protein–protein interaction partners, and essentiality for viability) expressed along early to late developmental stages (Irie and Sehara‐Fujisawa 2007; Roux and Robinson‐Rechavi 2008; Comte et al. 2010; Domazet‐Lošo and Tautz 2010; Yang et al. 2018). However, conclusions often were later labeled as controversial, as neither the evolutionary conservation of the embryonic phase nor the limiting effects on phenotypic diversification were unequivocally evident from the gene properties analyzed in these studies.

In this context, the cross‐species comparative transcriptomic approach was welcomed for its ability to assess commonality among developmental stages of different species, in particular, the evolutionary conservation of developmental stages (Kalinka et al. 2010; Irie and Kuratani 2011; Yanai et al. 2011; Wang et al. 2013; Hu et al. 2017; Zalts and Yanai 2017; Li et al. 2018, 2020; Marlétaz et al. 2018; Hogan et al. 2020; Chan et al. 2021; Uchida et al. 2022). This whole‐embryonic comparative transcriptomic approach, assumed to reflect the similar composition of homologous cells, has indicated a highly conserved mid‐embryonic period, or an hourglass‐like divergence in various animal groups, including chordates (Irie and Kuratani 2011; Yanai et al. 2011; Wang et al. 2013; Hu et al. 2017; Marlétaz et al. 2018), *Drosophila* species (Kalinka et al. 2010), *Caenorhabditis elegans* (Zalts and Yanai 2017), and echinoderms (Li et al. 2020). Consistent with the assumption of this approach, the highly conserved mid‐embryonic phase identified in vertebrates exhibits anatomical features that have been long conserved in vertebrates (Benton 2004; Hu et al. 2017), including the notochord, dorsal nerve cord, head with nostril, eye, ear, muscle blocks, pharynx with slits, trunk with the heart, liver, stomach, gonad, kidney, and tail with anus. However, the phylotype hypothesis of the hourglass model awaits further testing; whether the highly conserved mid‐embryonic phase represents the evolutionarily conserved basic anatomical pattern of each animal phylum, or body plan, remains unclear. For example, while the highly conserved stages of vertebrates give rise to their common anatomical features, it still remains to be tested if this also holds good for a wide range of species in the phylum Chordata. In echinoderms, the highly conserved developmental stages did not coincide with the phase when the body plan of echinoderms (pentameral body plan) begins to become evident, but occurred earlier during gastrulation (Li et al. 2020). While the discrepancy between the most conserved stages and the body plan can be regarded as evidence against the phylotype hypothesis, it may instead reflect problems in the definition of the body plan and phylum. In fact, although the body plan is defined as the set of morphological traits shared within a given phylum, the phylum itself is often defined in a circular manner as a monophyletic group of animals that share the same body plan (Irie et al. 2018).

## 2 Possible Limiting Effects of Embryogenesis on Phenotypic Diversification

The next question that arises is whether the transcriptomically conserved mid‐embryonic phase can be regarded as an outcome of developmental bias, or more specifically its limiting effect on diversifications. Studies so far do not appear to contradict this, while it may not be the sole cause of the hourglass‐like conservation during evolution.

One proposed idea is that the mid‐embryonic conservation could be explained by reduced diversifying selective pressures on traits expressed during the conserved mid‐embryonic period compared to earlier and later stages (Slack et al. 1993). Although this hypothesis was originally framed primarily from a developmental perspective, it has been supported by evidence that *cis*‐regulatory regions active during the mid‐embryonic period in two *Drosophila* species exhibit both inter‐species conservation and signs of depletion of positive selection in their sequences (Liu et al. 2021). However, the hypothesis attributing mid‐embryonic conservation solely to a lack of positive selection may not be sufficient. Zalts and Yanai (2017) demonstrated that the experimental evolution of *C. elegans* without positive selection (using mutation accumulation or MA line) also resulted in fewer phenotypic variations at the mid‐embryonic period among the experimentally evolved lineages, implying that other factors or mechanisms are required to explain the fewer variations (conservation) observed at the mid‐embryonic phase. Notably, considering that the MA line condition rapidly accumulates mutations, the persistence of relatively low transcriptomic variation observed during mid‐embryogenesis is consistent with the possibility that certain intrinsic properties of *C. elegans* developmental systems constrain or buffer the effects of accumulated mutations. In this context, one attractive hypothesis was that the mid‐embryonic phase is subject to stronger negative selection, with perturbations during this phase more likely to result in embryonic lethality than those occurring at earlier and later stages (Sander 1983; Raff 1996; Galis and Metz 2001). This “lethality hypothesis” appeared to align well with the meta‐analysis report that teratogens frequently target the mid‐embryonic organogenesis phase, disrupting normal development (Galis and Metz 2001).

However, an empirical test in three vertebrate species (zebrafish, African clawed frog, and chicken) did not support this idea (Uchida et al. 2018). In this study, embryos at different developmental stages were subjected to both environmental perturbations and genetic mutations. The results showed that embryonic lethality and severe malformations were not the highest when transient environmental perturbations were applied during the mid‐embryonic phylotypic period; instead, the strongest effects were observed when perturbations were applied earlier, during the gastrulation phase. Similarly, under mutation‐induced conditions, survival rates remained comparatively stable during the phylotypic period across the analyzed species, while the greatest decrease in viability was again observed around gastrula. Several critical caveats, however, complicate the interpretation of these lethality data, which Uchida et al. (2018) did not fully address. For instance, perturbations were applied at only a single, relatively severe level, reaching the median lethal dose (LD_50_), instead of applying a graded range of severities, and the fitness of embryos that survived without obvious lethal phenotypes was not quantified. Furthermore, the lethality rate at any given stage cannot by itself serve as a direct measure of the strength of negative selection by lethality against that stage, a difficulty compounded by at least two sources of temporal asynchrony between developmental disruption and observable lethality. First, if a mutation causes embryonic death at an early stage, it remains possible that the same mutation would have generated lethal phenotypes at later stages had the embryo survived, an outcome that cannot be assessed once embryogenesis has already been terminated. Second, conversely, disruptions to processes initiated at one stage may not produce overt lethality until considerably later in ontogeny. For example, Duboule (1994) has emphasized that fundamental body‐plan determinants such as Hox gene patterning and the segmentation clock are actively initiated during gastrulation; perturbations to these processes may therefore generate fitness costs that only fully manifest during or after the subsequent phylotypic period. Future studies should therefore assess not only stage‐specific embryonic survival, but also the long‐term reproductive consequences of perturbations applied at each developmental stage.

The caveats described above converge on a broader point: selection operates across the entirety of ontogeny, and the selective consequences of developmental perturbations can only be fully understood in the context of whole‐organism fitness across developmental stages. This perspective is also closely linked to another hypothesis proposed by Galis and Metz (2007), namely pleiotropic constraints. They proposed a more specific mechanism for how certain developmental processes can become evolutionarily conserved by their coupling to seemingly unrelated processes, including those occurring at other developmental stages. Pleiotropic constraints can arise when multiple developmental processes are coupled together. Such coupling may occur, for example, when pattern formation in different tissues depends on shared mechanical interactions or inductive molecular signals, or, at the subcellular level, when distinct biological processes rely on overlapping genetic systems. In these cases, perturbations to one process can propagate to others, resulting in phenotypic changes across multiple traits. Because such coupled developmental processes are jointly exposed to selection, changes affecting one component can have impacts on fitness through their effects on other traits, thereby constraining evolutionary divergence and potentially contributing to the conservation of the underlying developmental architecture. The effects of pleiotropy on evolution have been demonstrated through multiple lines of evidence, including similar expression patterns across different salmonid fish species (Papakostas et al. 2014) and limited beak diversity (Fritz et al. 2014). Conservation of serially homologous structures, such as butterfly wing eyespots (Allen et al. 2008), can also be regarded as consistent with pleiotropic constraints, since these structures often share extensive genetic and regulatory components and tend to evolve in a coordinated and interdependent manner, rather than independently (Allen et al. 2008; Young et al. 2010). Regarding the potential involvement of pleiotropic constraints in the mid‐embryonic conservation, one study examining genes expressed across developmental stages provided evidence consistent with this possibility (Hu et al. 2017). This study revealed that pleiotropically expressed genes (i.e., genes expressed across a variety of developmental stages) are more prevalent during the conserved phylotypic period than at other developmental stages in the vertebrate species analyzed (Figure 1B). Developmental stages enriched for such genes were associated with greater evolutionary conservation (Hu et al. 2017). In addition, the pleiotropically expressed genes found to be prevalent during the conserved phylotypic period in the study were also predicted to have many protein–protein interaction partners (Hu et al. 2017). This pattern appears conceptually aligned with Raff's earlier hypothesis that the phylotypic stage is characterized by extensive interconnection and global signaling networks among organ primordia, although that hypothesis was not specifically formulated in terms of protein–protein interactions (Raff 1996). Nevertheless, direct empirical evidence supporting this hypothesis or elucidating its relationship to the observed gene expression patterns is still lacking. Interestingly, beyond empirical findings, a simplified genetic model of development by Kohsokabe et al. (2024) also showed the accompanying increase in the fraction of pleiotropically expressed genes with the conserved period at the middle of development, despite unknown mechanisms.

An intriguing aspect of hourglass‐like conservation is that the phylotypic period in vertebrates is conserved not only at the macroevolutionary scale of the phylum or subphylum (Hu et al. 2017; Marlétaz et al. 2018), but also at finer evolutionary scales, including intra‐species level and even among individuals sharing minimal standing genetic variation (Wang et al. 2013; Uchida et al. 2022, 2023). By comparing whole‐embryonic transcriptomes of gender‐matched sibling embryos from a highly inbred medaka (Japanese rice fish) line raised under identical environmental conditions, Uchida et al. (2022) observed reduced gene expression variation during all candidate phylotypic periods (Figure 1C). These findings are consistent with the theoretical prediction that less stochastically fluctuating phenotypes, or phenotypes showing developmental stability—the tendency for a given genotype to follow the same developmental trajectory under identical conditions (Hallgrímsson et al. 2002)—tend to be evolutionarily conserved (Sato et al. 2003; Kaneko and Furusawa 2006). These findings collectively suggest that developmental stability may itself represent an intrinsic property of developmental bias, which could have contributed to the reduced phenotypic variability and evolutionary conservation observed at the mid‐embryonic period.

## 3 Remaining Questions and Challenges

**Figure 2:**
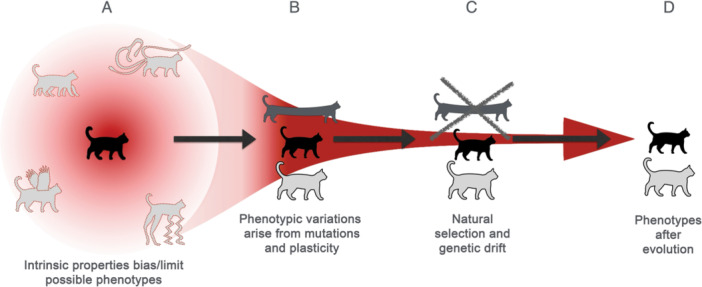
Possible bias by intrinsic properties toward phenotypic diversification. A schematic illustration of a hypothesis on how intrinsic properties can bias phenotypic evolution (A). Intrinsic biases, such as embryonic lethality, robustness, developmental stability, and pleiotropic constraints, limit the range of theoretically possible phenotypes that can emerge (B). During and following embryogenesis, mutations and phenotypic plasticity allow organisms to generate phenotypic variations (C). These phenotypic variations will be selected positively and negatively by natural selection as well as the effects of population genetics, which leads to phenotypic evolution (D). [Color figure can be viewed at wileyonlinelibrary.com]

Altogether, the mid‐embryonic, organogenesis period exhibits both a low potential to generate phenotypic variation regardless of the presence or absence of mutations, reflecting its intrinsic developmental robustness and developmental stability, and a marked enrichment of pleiotropically expressed genes whose broad activity across multiple biological processes may further restrict the range of accessible phenotypic outcomes. These observed intrinsic properties raise the possibility that the filtering or limiting aspect of embryogenesis toward phenotypic diversification (Figure 2) may not be solely implemented by selective pressures but also by intrinsic properties inherent to the organism or developmental program itself. However, considerable caution is needed before these intrinsic properties can be interpreted as the underlying mechanisms responsible for the long‐term evolutionary conservation of the mid‐embryonic phase.

Given the geometric properties of high‐dimensional genotype–phenotype maps, the fitness landscape of development is in principle highly navigable, and true fitness valleys are rare over macroevolutionary timescales (Gavrilets 1997; Greenbury et al. 2022). This implies that, given sufficient evolutionary time, no set of genotypes or phenotypic states can be completely confined by passive buffering mechanisms in their common ancestor alone. One consequence of this is that phenotypes currently regarded as inaccessible through a given embryonic developmental program, whether due to lethality or other constraints, may nonetheless become accessible over longer evolutionary timescales through the gradual remodeling of the developmental system itself. For example, the common ancestor of eukaryotes may have never given rise to primate‐like features as one of their phenotypic variations; however, through long‐term evolution, such traits eventually arose in their descendants, including humans. Therefore, although developmental robustness and stability can limit phenotypic diversity on shorter evolutionary timescales by reducing the rate at which heritable phenotypic variation is produced, they can be theoretically insufficient to confine morphological evolution against change over macroevolutionary timescales. Consistent with this reasoning, it has been suggested that robustness can also facilitate phenotypic diversification over long evolutionary timescales through the accumulation of cryptic genetic variations (Rutherford and Lindquist 1998; Shaw and Chang 2006; Ciliberti et al. 2007; Draghi et al. 2010; Wagner 2012; Siegal and Leu 2014). The molecular chaperone Hsp90, for instance, has been shown to buffer the phenotypic expression of a broad spectrum of underlying genetic variation in *Drosophila*, such that loss‐of‐function mutations reveal a diverse range of otherwise cryptic morphological phenotypes (Rutherford and Lindquist 1998). It is possible that the robust mid‐embryonic phase in vertebrates similarly conceals substantial cryptic variation, which could be phenotypically exposed if this robustness were compromised by mutation or environmental stress. Furthermore, while developmental robustness and stability have been observed at the vertebrate phylotypic period in extant species, it is no longer possible to directly study these properties in extinct ancestors in which the phylotypic period first became conserved, nor to determine whether these properties were continuously maintained since then. Therefore, it cannot be ruled out that robustness and stability are not the causes but rather the consequences of long‐term evolutionary selection pressures.

Similarly, the evolutionary role of pleiotropy requires further investigation, and has yet to be fully understood. Accumulating evidence demonstrates that gene co‐option and the rewiring of pleiotropic gene regulatory networks have been drivers of evolutionary novelty across animal lineages. Aves would be an example of this. Genomic sequences uniquely conserved across avian species were found almost exclusively in noncoding regions, indicating that the recruitment and rewiring of gene regulatory networks, rather than changes to protein‐coding sequences, played a central role in the diversification of novel traits in this lineage (Seki et al. 2017). Similarly, in mammals, the evolution of flight ability has been attributed in part to the co‐option of existing developmental programs (Feigin et al. 2023; Schindler et al. 2025). These examples suggest that pleiotropy may act as a double‐edged sword in phenotypic evolution, and whether and how the observed enrichment of pleiotropically expressed genes at the mid‐embryonic period specifically contributes to its conservation, therefore, remains a question requiring further investigation.

A further remaining question concerns how the intrinsic properties discussed above are mechanistically implemented at the molecular and cellular levels within the conserved mid‐embryonic period. Clarifying these mechanisms would not only explain how developmental robustness, stability, and pleiotropy arise and are maintained during the mid‐embryonic organ‐forming period, but would also provide a more direct basis for evaluating whether and to what extent they causally contribute to the long‐term evolutionary conservation of this period. Recent studies have begun to provide limited but suggestive insights. In *Drosophila*, the transcriptomic stability of the phylotypic period appeared to be associated with a distinctive pattern of histone modifications at developmental gene loci, suggesting a potential epigenetic basis for the stabilization of mid‐embryonic gene expression (Liu et al. 2020). In Japanese medaka, analysis of axial pattern‐forming genes suggested that the stability of whole‐embryonic gene expression during the mid‐embryonic period may be attributable to intracellular gene expression stability (Uchida et al. 2024). However, these insights remain indirect and mechanistically ambiguous, and the underlying molecular and cellular bases of the intrinsic properties of the mid‐embryonic period have yet to be systematically characterized. Recent technological advances, particularly in single‐cell and spatially resolved omics, offer promising avenues for addressing these gaps. By measuring the strength of these intrinsic properties and evolutionary conservation at the resolution of individual organ modules and cell types, it may become possible to identify which cellular compartments are more conserved and to begin dissecting the molecular architectures responsible for this conservation (Ma and Zheng 2024). Furthermore, as discussed above, testing whether highly interconnected signaling networks among embryonic tissues are indeed present during the conserved mid‐embryonic phase would be a first step toward evaluating whether inter‐organ interactions actually cause constraints on evolutionary change (Raff 1996). Although this hypothesis has long been discussed and cited as a potential mechanistic basis for pleiotropic constraints, direct empirical testing has remained largely lacking, mainly due to the technical difficulty of characterizing embryo‐wide signaling interactions with sufficient spatial resolution. Spatial transcriptome technology may now enable the systematic reconstruction of signaling networks among organ primordia with spatial context preserved, offering a path toward testing of this long‐standing hypothesis. Given the breadth of regulatory, cellular, and potentially mechanical factors likely to be involved, the mechanistic basis of these intrinsic properties is highly likely multifactorial, and the combinatorial interactions among contributing factors may not be fully captured by traditional methods based on pairwise correlations or single‐factor perturbation approaches. In this regard, machine learning models, which excel at extracting complex many‐to‐many relationships from high‐dimensional data, may represent a particularly valuable analytical framework for disentangling the contributing factors and their interactions (Eraslan et al. 2019). In particular, advances in interpretable “white‐box” modeling methods hold promise for identifying key developmental architectures underlying phenotypic conservation in a manner that remains both biologically interpretable and capable of generating experimentally testable hypotheses (Leong et al. 2025).

Although this review has focused primarily on the relationship between development and evolution, it is important to recognize that evolution is a multilayered process involving reciprocal interactions among genetics, development, ecology, and the environment, and that selection ultimately operates on whole organisms across their full life histories rather than on developmental stages in isolation. Understanding the intrinsic mechanisms of developmental bias at the phylotypic period is therefore a necessary but not sufficient step. How these mechanisms are integrated with population‐level processes, ecological pressures, and environmental changes must also be elucidated before the developmental insights here can be meaningfully formulated and incorporated into the broader framework of the Extended Evolutionary Synthesis (Laland et al. 2015). Bridging these scales of biological organization, from molecular mechanisms within individual embryogenesis to evolutionary dynamics across populations and lineages, represents one of the most fundamental challenges ahead of evolutionary developmental biology.

## Data Availability

Data sharing is not applicable to this article as no data sets were generated or analyzed during the current study.
